# *Streptococcus intermedius* lung abscess in children: a case report and literature review

**DOI:** 10.3389/fmed.2026.1948468

**Published:** 2026-09-11

**Authors:** Shuteng Ou, Shuhua Chen, Ting Wang, Biqing Zhang, Chuping Fan, Yandong Li

**Affiliations:** Department of Pediatrics, The First People’s Hospital of Chenzhou, Chenzhou, China

**Keywords:** children, global developmental delay, lung abscess, refractory epilepsy, streptococcus intermedius, targeted next-generation sequencing

## Abstract

*Streptococcus intermedius* is a gram-positive commensal associated with suppurative infections. Pediatric pulmonary abscesses due to *S. intermedius* are rare, and outcomes in children with severe neurological comorbidities are poorly characterized. A 5-year-old boy with refractory epilepsy and global developmental delay who presented with a right upper lobe abscess, pneumonitis, and pleural effusion. Targeted next-generation sequencing (tNGS) of bronchoalveolar lavage fluid (BALF), drainage pus culture, and tNGS of drainage pus all confirmed the presence of *S. intermedius*. To our knowledge, this is the first reported case of a residual pulmonary cavity on 3-month follow-up imaging after an *S. intermedius* lung abscess in a child with severe neurological impairment. Our findings highlight the value of tNGS in diagnosing lower respiratory tract infections caused by pathogens that are difficult to identify through conventional culture methods.

## 1 Introduction

*Streptococcus intermedius* is a gram-positive commensal bacterium of the human oropharynx. Although it is a low-virulence organism in immunocompetent hosts, it can cause severe suppurative infections, including brain abscesses, liver abscesses, endocarditis, and empyema, particularly in immunocompromised or neurologically impaired individuals (1). Pulmonary abscesses caused by *S. intermedius* are relatively uncommon, especially in the pediatric population. Most previously reported cases of *S. intermedius* lung abscess have occurred in adults with underlying conditions such as diabetes mellitus, malignancy, or periodontal disease (2, 3). In children, the literature consists solely of isolated case reports the majority of which have described previously healthy children without significant comorbidities (4–6).

However, the clinical course and outcome of *S. intermedius* lung abscess in children with severe neurological comorbidities, such as refractory epilepsy and global developmental delay, remain poorly characterized. It is unknown whether the presence of such comorbidities influences disease severity, treatment response, or radiological recovery.

To address this knowledge gap, we report a pediatric case of *S. intermedius* lung abscess in a child with refractory epilepsy and global developmental delay, along with a literature review of all previously reported pediatric cases. By comparing the clinical features, management, and outcomes of the neurologically impaired patient with those of the four neurologically intact children, we aim to highlight the impact of underlying neurological comorbidities on prognosis and to provide practical diagnostic and therapeutic recommendations for this vulnerable population.

## 2 Case presentation

A 5-year-old boy with a known history of refractory epilepsy and global developmental delay presented to our department with a 5-day history of persistent fever and cough. His seizures had been poorly controlled with antiepileptic drugs, manifesting as daily episodes of upward eye deviation with staring, without limb convulsions. He had previously undergone rehabilitation therapy at our hospital. At presentation, he was unable to sit independently, required full assistance with all activities of daily living, and showed significant delays in growth, development, and intellectual function compared with his peers. He had a history of multiple hospitalizations for pneumonia. There was no history of surgery, trauma, or dental procedures. All scheduled vaccinations, including BCG, were administered. The family history was negative for asthma, diabetes mellitus, immunodeficiency, malignancy, and tuberculosis.

**Figure 1:**
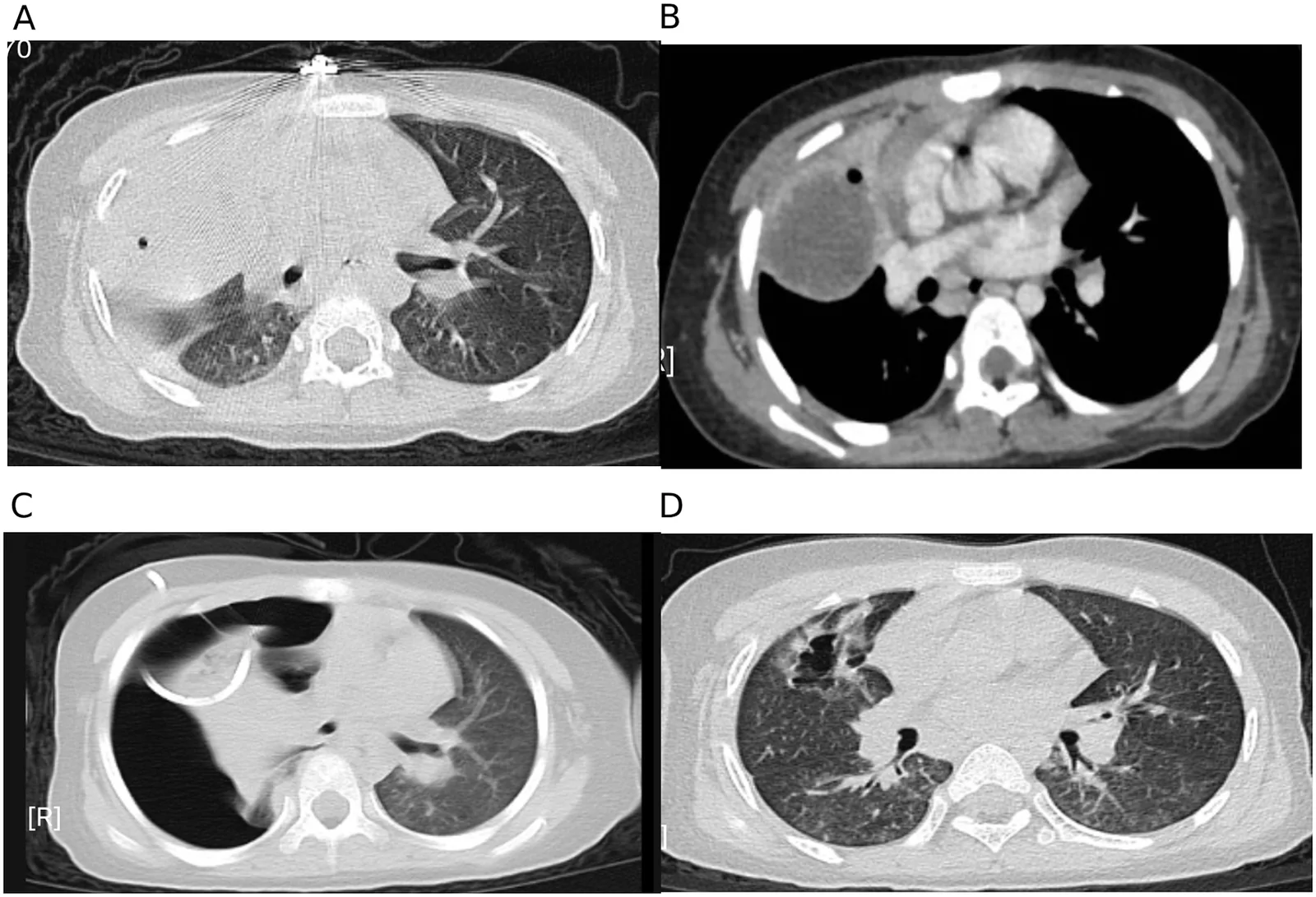
Serial chest CT images of the patient. **(A)** Chest CT images obtained at presentation demonstrate a large patchy high-density opacity (68 mm × 46 mm) in the right upper lobe with a small pleural effusion. **(B)** Contrast-enhanced chest CT images obtained after 11 days of treatment demonstrate partial resolution of the right upper lobe mass-like opacity (58 mm × 39 mm). **(C)** Pneumothorax on day 5 after ultrasound-guided percutaneous drainage. **(D)** Follow-up chest CT images obtained 3 months after discharge show a cavity (22 mm × 15 mm) along the right horizontal fissure.

On the third day of fever, he visited a local hospital where laboratory tests revealed a white blood cell count of 29.6 × 10^9^/L with 69.4% neutrophils, a hemoglobin concentration of 119 g/L, a platelet count of 318 × 10^9^/L, and a C-reactive protein concentration of 86.9 mg/L. He was initially treated with oral cefixime for three days without improvement and was subsequently transferred to our hospital at his parents' request. On arrival, his vital signs were as follows: body temperature, 38.7 °C; pulse, 119 beats per minute; respiratory rate, 30 breaths per minute; and weight, 14 kg. Physical examination revealed decreased breath sounds on the right side. Admission laboratory investigations are summarized in Table 1. Tests for HIV, hepatitis B surface antigen, anti-hepatitis C virus antibody, syphilis, serum (1,3)-β-D-glucan, and galactomannan were all negative. Given concerns about endocarditis or other occult sources of infection, cardiac and abdominal ultrasonography was performed and revealed no abnormalities. On hospital day 1, chest computed tomography (CT) revealed a right lung infection with a large patchy high-density opacity in the right upper lobe and a small pleural effusion (Figure 1A). Empirical antibiotic therapy with intravenous ceftriaxone and vancomycin was initiated.

**Table 1:** Laboratory tests on admission.

Laboratory test	Results of case	Reference value
WBC (×10^9^/L)	28.9	4.4–11.9
Neutrophil count (×10^9^/L)	19.6 (67.6%)	1.2–7.0
Lymphocyte count (×10^9^/L)	6.6 (22.7%)	1.8–6.3
Monocyte count (×10^9^/L)	2.54 (8.8%)	0.12–0.93
Hemoglobin (g/L)	106	112–149
Platelet count (×10^9^/L)	443	118–472
C-reactive protein (mg/L)	73.8	<8
Procalcitonin (ng/mL)	0.1	0–0.5
ESR (mm/h)	64	0–15
Blood coagulation function test	Normal	Normal
D-dimer (mg/L)	2.3	0–0.55
Immunoglobulin testing (5 items)	Normal	Normal
Renal function	Normal	Normal
Myocardial enzyme	Normal	Normal
Liver function	Normal	Normal
Albumin (g/L)	31.4	39–54
Respiratory tract pathogen PCR (13 items)^a^	Negative	Negative
T-SPOT.TB, PPD	Negative	Negative
Acid-fast staining smear(BALF)	Negative	Negative
Gram-stained smear(BALF)	positive (Gram-positive cocci)	Negative
Blood culture	Negative	No growth
Sputum culture	Negative	No growth
BALF culture	*Streptococcus viridans group* (scanty)	No growth
Drainage pus culture	*Streptococcus intermedius*	No growth
tNGS detected *S. intermedius* in BALF	33,707 genetic reads	Negative^c^
tNGS detected *S. intermedius* in drainage pus	192,156 genetic reads^b^	Negative

a The 13 respiratory tract pathogens included: Influenza A virus, Influenza A H1N1, Seasonal H3N2, Parainfluenza virus, Human Metapneumovirus, Mycoplasma pneumoniae, Respiratory syncytial virus, Adenovirus, Rhinovirus, Bocavirus, Chlamydia pneumoniae, Influenza B virus and Coronavirus.

b The *tetM* gene was detected in 94 sequences. The presence of this gene confers resistance to tetracycline antibiotics.

c Positive criteria: RPM (Reads Per Million) >50 or RPM >20 with ≥2 primer pairs.

WBC, white blood cells; ESR, erythrocyte sedimentation rate; BALF, bronchoalveolar lavage fluid; T-SPOT.TB, tuberculosis-specific enzyme-linked immunospot Assay; PPD, purified protein derivative; PCR, polymerase chain reaction; tNGS, targeted next-generation sequencing.

On hospital day 3, the patient became afebrile and underwent flexible bronchoscopy with bronchoalveolar lavage (BAL). The BAL fluid findings are presented in Table 1. However, on hospital day 11, contrast-enhanced chest CT showed a lesion in the right upper lobe that was partial resolution from the previous scan (Figure 1B). During hospitalization, he experienced one seizure episode. On day 12, a multidisciplinary consultation involving neurology, thoracic surgery, pharmacy, and ultrasound specialists was conducted. On day 13, laboratory tests revealed a white blood cell count of 17.4 × 10^9^/L with 61.3% neutrophils, a hemoglobin concentration of 111 g/L, a platelet count of 363 × 10^9^/L, and a C-reactive protein concentration of 6.3 mg/L. The antibiotics were changed to intravenous amoxicillin-clavulanate. Ultrasound-guided percutaneous drainage of the lung abscess was subsequently performed, yielding approximately 10 mL of thick yellow purulent fluid. The aspirated fluid was sent for cytology, biochemistry, Gram staining, and microbiological studies. The fluid was yellow and turbid and without clotting; the red blood cell count was 10 × 10^6^/L, and the nucleated cell count was markedly elevated (+++ × 10^6^/L), with 95% polymorphonuclear cells, 5% mononuclear cells, and 5 pus cells per high-power field. Gram staining of the drainage fluid revealed gram-positive cocci. Targeted next-generation sequencing (tNGS) of the drainage fluid revealed *Streptococcus intermedius*, yielding 192,156 sequencing reads. Four days later, both aerobic and anaerobic cultures of the drainage fluid consistently yielded *S. intermedius*, and antimicrobial susceptibility testing demonstrated susceptibility to Vancomycin, Meropenem, Daptomycin, Linezolid, Penicillin, Ampicillin, Ceftriaxone, Levofloxacin (Table 2).

**Table 2:** Antimicrobial susceptibility profile of the *Streptococcus intermedius* isolate.

Antimicrobial agent	MIC (*μ*g/mL)	Interpretation^a^
Clindamycin	≧2	R
Vancomycin	≦0.5	S
Meropenem	≦0.25	S
Daptomycin	≦1	S
Linezolid	≦2	S
Penicillin	≦0.06	S
Ampicillin	≦0.25	S
Ceftriaxone	≦0.5	S
Tetracycline	≧16	R
Levofloxacin	≦2	S
Erythromycin	≧8	R

a S, susceptible; I, intermediate; R, resistant. MIC breakpoints were interpreted according to CLSI M100, 35th edition (2025), using breakpoints for viridans group streptococci.

After ultrasound-guided percutaneous drainage, fever persisted for two additional days. On day 5 post-drainage, a pneumothorax occurred (Figure 1C). The drainage tube was promptly repositioned, and subsequent chest radiography confirmed resolution, following which the tube was removed. A chest radiograph was obtained on hospital day 25. The patient was subsequently discharged on a sequential oral antibiotic regimen consisting of amoxicillin-clavulanate for 2 weeks and linezolid for 2 weeks.

At follow-up, there was no abscess recurrence. The frequency of epileptic seizures did not increase. A repeat chest CT performed 3 months after discharge revealed a cavity in the right horizontal fissure region (Figure 1D).

## 3 Review of the literature

The clinical characteristics and outcomes of previously reported pediatric cases are summarized in Table 3.

**Table 3:** Clinical characteristics and outcomes of pediatric *S.intermedius* lung abscess cases reported in the literature.

Author, (year)	Age (years)	Sex	Underlying conditions	Clinical presentation	Chest CT findings	Microbiologic diagnosis	Antibiotic therapy	Other treatment	Outcome
Nakagawa et al., 2022 (4)	0.5	M	None	Fever, cough and dyspnea	Right cavity lesion and pleural effusion	Anaerobic culture of pleural fluid	Amoxicillin/clavulanate (8 weeks)	Pleural puncture, drainage and urokinase	Abscess resolved
Huang et al.,2022 (5)	10	M	None	Chest pain	Round soft tissue mass and rim-enhancing lesion in the left lower lobe	Lung biopsy tissue culture and mNGS	Amoxicillin/clavulanate (11d) → linezolid(37d)	Tracheoscopy lavage, pleural puncture and drainage	Abscess resolved
Xu et al., 2023 (6)	5.3	M	None	Fever, cough and chest pain	Right upper lobe abscess, peripheral pneumonitis and pleural effusion	Lavage fluid culture and mNGS	Ceftriaxone sodium (10d) → linezolid(10d) → amoxicillin/clavulanate (2d)	Tracheoscopy lavage	Abscess resolved
Xu et al., 2023 (6)	1.8	M	None	Fever and cough	Pneumonia with right encapsulated pleural effusion, right middle lung atelectasis and abscess	Pleural fluid mNGS	Ceftriaxone sodium (6d) → linezolid (15d)	Tracheoscopy lavage and pleural puncture/drainage	Abscess resolved
Present case	5	M	Epilepsy and global developmental delay	Fever and cough	Abscess in the upper lobe of the right lung with peripheral pneumonitis and pleural effusion	BALF tNGS, drainage pus culture and tNGS	Ceftriaxone and vancomycin (14d) → amoxicillin/clavulanate(25d) → linezolid (14d)	Tracheoscopy lavage and abscess puncture/drainage	Residual lung cavity

BALF, bronchoalveolar lavage fluid; CT, computed tomography; M, male; d, days; mNGS, metagenomic next-generation sequencing; tNGS, targeted next-generation sequencing.

## 4 Discussion

*Streptococcus intermedius* is a member of the *Streptococcus anginosus* group (SAG) (7), also known as the *Streptococcus milleri* group, which primarily includes *S. intermedius*, *Streptococcus anginosus*, and *Streptococcus constellatus* (8, 9). Among these, *S. intermedius* is most frequently associated with suppurative infections, particularly brain and liver abscesses (1, 10). However, lung abscesses caused by *S. intermedius* are rare (11) and are predominantly reported in adults, being exceedingly uncommon in children. The literature search was conducted in PubMed and Web of Science using the search terms “child,” “*Streptococcus intermedius*,” and “lung abscess.” To date, including the present case, only five pediatric cases of *S. intermedius* lung abscess have been reported (4–6). This remarkably low number underscores the rarity of the disease in the pediatric population. The clinical characteristics, treatment, and outcomes of all five patients are summarized in Table 3.

Among the five reported cases, the median age was 4.5 years (range: 0.5–10 years), and all the patients were male. Four of the five children (80%) were previously healthy, whereas one patient had refractory epilepsy and global developmental delay. These findings suggest that although *S. intermedius* lung abscess can occur in immunocompetent children without comorbidities and warrants clinical attention, severe neurological impairment may be a predisposing factor (12).

*S. intermedius* is a commensal of the oropharynx and produces a potent arsenal of tissue-destroying enzymes that promote abscess formation (1). Most notably, the primary virulence factor is intermedilysin (13), a cholesterol-dependent cytolysin that lyses human erythrocytes and nucleated cells (9). Additional enzymes including hyaluronidase, chondroitin sulfatase, and DNase, degrade extracellular matrix components and facilitate tissue invasion and cavity formation. In the present series, chest CT scans of all five children revealed large lesions with or without air-fluid levels, corroborating the tissue-destructive potential of the organism. Children with neurological comorbidities may be at an increased risk of lower respiratory tract infection because of poor neuromuscular coordination, ineffective cough reflex, uncoordinated swallowing, and poor oral hygiene (14). The route of infection in this case of *S. intermedius* remains unclear. We speculate that silent aspiration may be the likely mechanism. However, it must be emphasized that this speculation lacks direct evidence, as objective assessments such as videofluoroscopic swallowing study (VFSS), fiberoptic endoscopic evaluation of swallowing (FEES), or esophageal pH monitoring were not performed in this case. This speculation is mainly based on the following: (1) the patient had a history of refractory epilepsy, and recurrent seizures can lead to decreased consciousness and impaired airway protective reflexes, increasing the risk of aspiration; (2) *S. intermedius* is a common commensal organism of the oropharynx, and the anatomical route of inhalation into the lower respiratory tract is plausible; (3) imaging showed cavitation, which is consistent with the typical features of aspiration-related infection. Nevertheless, in the absence of objective swallowing/aspiration assessment, this mechanism should be regarded only as a reasonable explanatory hypothesis rather than a definitive conclusion. For future cases with similar clinical backgrounds, routine evaluation of swallowing function and aspiration risk is recommended to clarify the route of infection and guide the development of preventive intervention strategies.

Four of the five children (80%) presented with fever and respiratory symptoms, while one presented with respiratory symptoms alone (no fever). The youngest patient, an infant aged 0.5 years, presented with dyspnea (4). Notably, none of the patients exhibited classic signs of severe pneumonia (e.g., tachypnea, retractions, or hypoxemia) upon initial evaluation, a factor that may have contributed to diagnostic delay. Typical CT features of a lung abscess include a thick-walled cavity containing an air-fluid level, rim enhancement, surrounding inflammatory infiltration, and pleural reaction. In our case and in the reviewed literature (5), air-fluid levels were absent. This absence may be attributed to factors such as the hematogenous origin of the abscess, the early stage of the disease, the specific anatomical location (e.g., the apical segment) (15), or the specific pathogen involved (e.g., anaerobic *Treponema denticola)* (16).

Although conventional culture is considered the gold standard for pathogen identification, it has several limitations, including low positivity rates, a prolonged turnaround time, and difficulty in detecting atypical pathogens (e.g., viruses and mycoplasmas), particularly when analysing sputum or upper respiratory tract samples that are often contaminated by oral commensal flora (17). Routine aerobic sputum and blood cultures have notably low detection rates for *S. intermedius*, which frequently results in the organism being overlooked in clinical microbiology laboratories and, consequently, being underestimated as a cause of lung abscess (18). *S. intermedius* is typically classified as a microaerophilic coccus, but its growth is enhanced under anaerobic conditions, likely due to increased glucose metabolism and upregulated expression of genes involved in nucleoside and nucleotide metabolism (1). Some authors have recommended anaerobic culture of drainage fluid specimens (4), including blood cultures, particularly in pediatric cases of empyema, brain abscess, and liver abscess potentially caused by *S. intermedius*. Compared with conventional techniques, metagenomic next-generation sequencing (mNGS) enables the detection of all microbial nucleic acids (bacteria, viruses, fungi, and parasites) in clinical samples, which is especially valuable for identifying anaerobic bacteria, rare microorganisms, and polymicrobial infections. In the cases we reviewed, *S. intermedius* was successfully identified by mNGS of pleural fluid or lung biopsy tissue (5). Targeted next-generation sequencing (tNGS) enhances the amount and proportion of pathogen-derived data by upfront capture and enrichment of specific pathogen nucleic acids before high-throughput sequencing and bioinformatics analysis, thereby improving detection sensitivity (19). Therefore, tNGS may provide a novel, broad-spectrum, rapid, accurate, and cost-effective approach for the diagnosis of respiratory tract infections (20). Moreover, it can detect antimicrobial resistance genes, which may aid antimicrobial stewardship and the formulation of individualized treatment strategies. In this study, although the tetM gene was detected, it could not be attributed to a specific pathogen. The tNGS report noted that the potentially associated pathogen was *S. intermedius*, however, this association is not definitive. Moreover, discrepancies may exist between resistance genotypes and phenotypes, and detection of a resistance gene does not confirm that the corresponding organism is phenotypically resistant to the relevant antimicrobial agent. Therefore, this finding should be regarded as providing reference information only and should be interpreted by clinicians in the context of the actual clinical situation. In our case, the results of BALF tNGS, drainage pus culture, and drainage pus tNGS were positive for *S. intermedius*. Based on our case series and the literature review, we emphasize that tNGS of drainage fluid or bronchoalveolar lavage fluid is a valuable diagnostic tool, particularly when conventional cultures are negative or when invasive biopsy is highly risky (21, 22).

Most lung abscesses can be successfully treated with antibiotics alone. However, treatment failure can occur, potentially attributed to pathogen virulence, insufficient antibiotic concentrations within the abscess cavity, or severe underlying lung disease preventing spontaneous drainage of the cavity (23, 24). When conservative management fails, surgical resection is typically indicated. An alternative approach is drainage. The role of percutaneous catheter drainage (PCD) in the management of pyogenic lung abscesses remains controversial (23, 25), but it can shorten the duration of antibiotic therapy and the length of hospital stay (26). PCD is generally performed under fluoroscopic, ultrasound, or CT guidance to avoid traversing the unaffected lung. Catheter blockage is a common complication, and may be associated with the use of small-caliber catheters (25). Pneumothorax, hemothorax, and hemoptysis are potential complications. Therefore, the procedure requires a careful risk-benefit assessment and close observation of the child. Another option is endoscopic drainage (ED), which is considered an alternative to percutaneous drainage for patients with coagulopathy, airway obstruction, or more centrally located abscesses (23).

The four children without underlying comorbidities in this review had favourable outcomes after timely anti-infective therapy and drainage (PCD), which is consistent with the existing literature (27, 28). To the best of our knowledge, this is the first pediatric case report of *S. intermedius* lung abscess with pulmonary cavitation occurring in the setting of severe neurological deficits. The presence of severe neurological comorbidities may be associated with a poorer radiological prognosis, however, this finding is based on a single case and requires further validation in larger cohort studies. In cases of refractory lung abscesses, multidisciplinary management involving specialists in pediatrics, thoracic surgery, radiology, ultrasonography, and pharmacy is often needed to optimize treatment outcomes and reduce the risk of complications.

We have explicitly acknowledged several limitations of this study, including its single-case design, the extremely small number of comparable published pediatric cases, the inability to establish a causal relationship between neurological disease and prognosis, the absence of objective evidence for aspiration, and the inherent limitations associated with interpreting tNGS findings.

## 5 Conclusions

*S. intermedius* lung abscesses are relatively rare in children relative to abscesses at other sites. This condition can occur in previously healthy children, yet its pathogenicity is often underestimated. Following aggressive treatment, the prognosis is generally favourable. Neurological impairment could be a potential factor influencing recovery, though this remains a hypothesis that warrants future studies. This case report highlights the potential value of tNGS in the diagnosis of lower respiratory tract infections caused by difficult to identify pathogens, thereby providing evidence to support its clinical application.

## Data Availability

The original contributions presented in the study are included in the article/Supplementary Material, further inquiries can be directed to the corresponding author.
