# Supplementary material for: Streptococcus intermedius lung abscess in children: a case report and literature review
Source: Front Med (Lausanne). 2026 Sep 11;13:1948468. doi: 10.3389/fmed.2026.1948468 (PMC13612531; doi:10.3389/fmed.2026.1948468)
Supplement: Supplementary file 1 [file Table1.docx]

Supplementary Material

# 1 Supplementary Tables

| **Supplementary TABLE 1.** Timeline of patient’ s course of illness | | | | |
| --- | --- | --- | --- | --- |
| **Date** | **Symptoms onset** | **Test** | **Treatments administered** | **Follow-up outcomes** |
| 2025.12.4 | fever and cough | Chest CT | Ceftriaxone and vancomycin | Lung abscess |
| 2025.12.7 | absence of fever |  | Continue anti-infective treatment | Afebrile |
| 2025.12.8 |  | tNGS of BALF | Flexible bronchoscopy with BAL |  |
| 2025.12.15 |  | Contrast-enhanced chest CT |  | Partial resolution |
| 2025.12.17 |  | WBC decreased compared with the previous value | Changed to intravenous amoxicillin‑clavulanate | MDT |
| 2025.12.18 | fever for 2 days | Drainage pus culture and tNGS | Ultrasound-guided percutaneous drainage |  |
| 2025.12.23 |  | Chest CT |  | Pneumothorax |
| 2025.12.24 |  | DR |  | Reduced volume of pneumothorax |
| 2025.12.26 |  | DR | Tube thoracostomy removal | Cavitary lesion in the lung |
| 2025.12.27 | fever |  |  |  |
| 2025.12.28 | fever |  |  |  |
| 2025.12.29 | absence of fever | DR | De-escalated to oral amoxicillin-clavulanate for 2 weeks | The pneumothorax resolved and the patient was discharged |
| 2026.1.14 | Re-exacerbation of pre-existing cough | DR | Linezolid for 2 weeks | Cavitary lesion in the lung |
| 2026.4.28 |  | Chest CT |  | Cavitary lesion in the lung |
|  |  |  |  |  |
